# Algorithmic management and psychosocial risks at work: An emerging occupational safety and health challenge

**DOI:** 10.5271/sjweh.4270

**Published:** 2026-01-01

**Authors:** Mairi Bowdler, Heidi Lahti, Marie Jelenko, Giuliana Buresti, Teppo Valtonen

**Affiliations:** 1 Netherlands Organisation for Applied Scientific Research (TNO), Leiden, The Netherlands.; 2 Finnish Institute of Occupational Health, Helsinki, Finland.; 3 Faculty of Social Sciences, Tampere University, Tampere, Finland.; 4 Austrian Workers’ Compensation Board (AUVA), Vienna, Austria.; 5 INAIL - Italian Workers’ Compensation Authority, Rome, Italy.

In recent years, algorithmic management (ALMA) systems have spread rapidly from platform work to more traditional sectors such as logistics, retail, and healthcare (1, 2). Defined as “the use of complex digital systems or AI [artificial intelligence] to manage workers” (3) and encompassing the semi- and fully automated execution of managerial functions such as scheduling, goal setting, and performance evaluation (4), ALMA is profoundly reshaping how work is organized and monitored. Building on recent discussions in this journal on digitalization and occupational health (eg 5), this editorial examines how ALMA is transforming working conditions and power dynamics and considers its implications for workers’ health and well-being in changing work environments.

A review of the literature up to 2022 (6) synthesized emerging evidence on how ALMA affects job quality and worker health in platform work, highlighting psychosocial risks such as intensified workload, irregular and unpredictable working hours with unpaid waiting times, reduced decision authority, and social isolation. In other words, ALMA can recalibrate the balance between job demands and resources in ways that heighten psychosocial risks. Examined in the light of established models of psychosocial risk (7) – such as the Job Demands–Resources model (8, 9), the Job Demand–Control–Support model (10, 11), and the Effort–Reward Imbalance model (12, 13) – these prior findings suggest that ALMA can create conditions that trigger classic risk pathways in which job demands exceed available resources, leading to stress, burnout, and related health outcomes.

However, ALMA is not inherently detrimental. A growing body of research emphasizes that ALMA is not just a technical system, but a socio-technical process shaped by organizational contexts and human decisions (1, 4, 14). Thus, ALMA's implications for work and workers depend not only on technical features but also, for instance, how and for what purpose the systems are used. When designed with transparency, fairness, and the opportunity for human influence over the system (4), the harmful effects of ALMA might be mitigated. Some researchers have even underscored the dual effect of ALMA, noting that algorithmic systems can both constrain and enable autonomy and value to workers depending on their design (15). However, evidence for positive outcomes remains limited and largely theoretical (4). While some studies have pointed to potential benefits for workers, such as increased flexibility in deciding working hours, these are often accompanied by new forms of control (16).

This editorial builds on a structured (non-systematic) literature review conducted within the PEROSH network (3), which followed a systematic search process and was visualized according to PRISMA 2020 reporting principles (17). The review synthesized findings from 39 studies (published 2022–2024) on the Occupational Safety and Health (OSH) implications of ALMA, covering both traditional and platform work. While the resulting report itself was not peer-reviewed, it draws exclusively from high-quality empirical sources (33 peer-reviewed scientific studies and 6 grey literature reports) and was developed by a team of researchers across multiple European institutions (3). Based on the review, we argue that ALMA is not merely a digital enhancement of existing managerial practices. Rather, it constitutes a novel form of work organization that shifts managerial decision-making from humans to algorithms, thereby reshaping the psychosocial work environment and redistributing power, control, and responsibility. The following sections summarize the key findings of our review, discuss their implications for policy and practice, and outline research priorities to ensure that ALMA supports rather than undermines workers’ health and well-being.

## Key findings of the review

The ALMA-AI project’s review indicated that the use of ALMA systems frequently leads to excessive job demands while simultaneously reducing key job resources needed to manage those demands. This imbalance shapes working conditions in ways that heighten psychosocial pressures and increase the risk of adverse OSH outcomes. In the ALMA-AI report, these patterns were identified and subsequently structured into both quantitative and qualitative studies, spanning across platform work and traditional workplaces.

## Intensification of job demands

Quantitative analyses conducted in platform work settings consistently show that ALMA systems generate psychosocial – particularly time – pressures that significantly elevate work-related stress levels (eg 18–21,). Time pressure as a job demand was also referred to in qualitative studies that identified excessive workloads as a recurrent feature of ALMA across various platform sectors (eg 22, 23,). In some accounts, workers even described how these heightened demands made them feel “exploited” (24). Similar findings were highlighted in an ILO report, where the intensification of work appeared to be directly linked to the use of monitoring systems (25).

## Depletion of job resources

The review showed that the negative impact of ALMA on OSH is often exacerbated when these systems undermine key job resources; a pattern observed particularly when ALMA is used as a control mechanism. Such effects include reduced autonomy (26) and diminished social support, manifested for instance as limited time to interact with co-workers (25). ALMA often imposes standardized workflows, reducing opportunities for worker discretion (27). The loss of autonomy is especially pronounced when algorithms are perceived as opaque (26) or used to impose strictly timed or closely monitored tasks (28). Some of the reviewed studies also reported that workers frequently feel excluded from decision-making processes surrounding the introduction and use of ALMA systems (eg 25,).

## Empirical evidence from large-scale surveys

In addition to peer-reviewed studies, the review integrated findings from major institutional reports based on large, representative samples. The EU-OSHA report (29) drew on OSH Pulse survey data covering 27 250 workers across the EU. The results suggest that each one-unit increase in ALMA intensity was associated with a 21% rise in psychosocial risks and a 16.5% increase in health issues. Similarly, a Foundation for European Progressive Studies 2023 survey of 5141 workers in Nordic countries (30) found that intensive use of ALMA nearly doubled stress levels compared with workplaces without ALMA.

## Worker involvement and transparency as mitigation strategies

Another key finding of the review was the importance of potential “moderators” that can buffer the negative effects of ALMA. Two strategies appeared: worker involvement and transparency. In terms of worker involvement, collective worker representation has been effective in negotiating limits on algorithmic control, protecting worker privacy, and discretion (31). Participatory approaches, such as co-design and collective bargaining, can help ensure worker influence in the implementation of ALMA, supporting autonomy and trust (30).

While not as impactful as direct involvement, transparency also plays a key role in mitigating the negative effects of ALMA. Clearly communicating how algorithms function and how decisions are made can help maintain job satisfaction, motivation, and trust (30). Transparency also further enhances perceptions of fairness, particularly in platform work settings (21). These two strategies are well-established in OSH practice and remain vital as algorithmic systems evolve.

## Research and methodological implications

The findings of the review conducted in the ALMA-AI project underscore that ALMA systems often create an imbalance between job demands and available job resources, contributing significantly to psychosocial risks and negative OSH outcomes. While worker participation and transparency can help mitigate these effects, the novelty and complexity of ALMA call for continuous research, adaptive regulation, and collaboration across stakeholders.

Future research should focus on effective strategies to protect OSH under ALMA, with particular attention to moderating factors such as worker participation, transparency, and the broader socio-technical context. Longitudinal studies are particularly needed to assess the long-term effects of ALMA and capture adaptation processes over time.

A key methodological challenge concerns the lack of standardized and validated tools for assessing ALMA intensity, functions, and impacts, particularly in traditional workplaces. Existing instruments, such as the Algorithmic Management Questionnaire (AMQ) (32) provide a valuable foundation for measuring ALMA exposure and its OSH implications. However, a universally accepted methodology for internal risk assessment is still lacking. The AMQ requires further validation, translation, and adaptation across countries, sectors, and employment types. Some items developed for platform work, such as those related to compensation or job termination, may be less relevant in traditional organizations, whereas new dimensions, like algorithmic task allocation, may have significant psychosocial relevance.

Developing robust and context-sensitive assessment tools capable of capturing the intensity, functions, and uses of ALMA systems and related practices is a fundamental research priority. Such tools would support both scientific understanding and policy development by providing a clearer basis for practice-oriented regulation and workplace risk assessment. Importantly, future studies should account for differences between platform and traditional workplaces, where ALMA systems are embedded within existing managerial structures and practices (1).

Continued research is essential to refine and expand measures that safeguard workers’ rights and well-being in increasingly digital workplaces, ensuring that ALMA systems align with principles of health, safety, and fundamental rights such as privacy, equality, and non-discrimination.

## Policy and practice implications

The findings of the ALMA-AI review (3), together with prior research (6), demonstrate that ALMA fundamentally reshapes power relations at work, with control shifting increasingly from workers to employers and platform operators. Of particular concern is the growing risk of psychosocial hazards. These risks often remain obscured, as ALMA systems may be perceived as objective or neutral due to their technical logic. In practice, however, these systems are designed for specific organizational purposes and reflect the values and assumptions of their developers and implementers. Software developers, therefore, represent an additional stakeholder group in OSH, as their design choices may consciously or unconsciously embed management logics.

The impact of ALMA on workers ultimately depends on managerial choices and the broader organizational context. In many cases, employers and platform operators prioritize efficiency and profit, leading to intensified control over workers’ tasks and conditions (33, 34). This underscores the need for robust regulatory and organizational safeguards that ensure compliance with OSH standards and the protection of workers’ health and rights.

A significant step in this direction has been taken with the adoption and forthcoming implementation of the EU Artificial Intelligence Act. The classification of AI systems used in worker management as “high risk” requires employers to conduct comprehensive assessments and mitigate potential impacts on OSH and fundamental rights. However, legal safeguards alone cannot address the complex organizational and psychosocial dynamics documented in empirical studies (eg 4,). OSH research therefore remains crucial in informing both regulation and workplace practice.

From a policy and practice perspective, regulations should mandate OSH risk assessments for ALMA systems, whether or not they include AI. These assessments must safeguard privacy, equality, and non-discrimination, and ensure workers’ right to information and transparency in how such systems operate. Evidence from the ALMA-AI review shows that worker participation and transparency in algorithmic processes are decisive in mitigating negative impacts. Therefore, employers should adopt participatory design and consultation processes when introducing ALMA systems and ensure accountability in algorithmic decision-making. ALMA-related risks should also be incorporated into occupational risk management systems and complemented by training on psychosocial risk prevention.

Finally, a coordinated European effort involving OSH institutions, researchers, policymakers, and social partners is essential to monitor and manage the psychosocial risks associated with ALMA. Such cooperation should ensure meaningful human oversight, strengthen transparency, and enhance workers’ capacity to engage in dialogue over system design and implementation.
